# Implications of LncRNAs and CircRNAs in psoriasis: a review

**DOI:** 10.1080/15476286.2023.2223486

**Published:** 2023-06-13

**Authors:** Rongcan Shi, Rui Ma, Xingyu Jiang, Xinyi Tang, Yu Gong, Zengyang Yu, Yuling Shi

**Affiliations:** a Department of Dermatology, Shanghai Skin Disease Hospital, Tongji University School of Medicine, Shanghai, China; b Department of Dermatology, Shanghai Tenth People’s Hospital, Tongji University School of Medicine, Shanghai, China; c Institute of Psoriasis, Tongji University School of Medicine, Shanghai, China

**Keywords:** Psoriasis, LncRNA, CircRNA, Keratinocyte, Immune cell

## Abstract

Psoriasis is a chronic inflammatory skin disease characterized by skin infiltration of immune cells and abnormal epidermal thickening. The initial pathogenesis has not been fully elucidated. Non-coding RNAs (ncRNAs), which include long ncRNAs (lncRNAs) and circular RNAs (circRNAs), comprise the majority of genome transcripts and are important influencers of gene transcription and post-transcription modulations. Emerging roles of ncRNAs in psoriasis were identified recently. This review summarizes the existing studies of psoriasis-related lncRNAs and circRNAs. A considerable proportion of the studied lncRNAs and circRNAs regulate keratinocyte mobility, such as keratinocyte proliferation and differentiation. Some lncRNAs and circRNAs are tightly related to keratinocyte inflammation reactions. Other reports demonstrated that they are also implicated in modulating immune cell differentiation, proliferation, and activation. This review might illuminate future psoriasis research and highlight that lncRNAs and circRNAs might act as therapeutic targets.

## Introduction

Current research progress confirmed that psoriasis is a systematic and complex disease and presently cannot be cured. Although the application of biological agents that target IL−17A, IL−23, or TNF-α significantly improved the treatment of psoriasis, they cannot completely eliminate the disease [1]. Therefore, there remains an urgent need to explore the initial pathogenesis of psoriasis. Genetic susceptibility is tightly related to psoriasis risks, particularly in the presence of the HLA-C × 06:02 risk allele [2,3]. Comprehensive assessment of psoriasis from a genetic perspective (including coding and non-coding genes) is necessary. Given that the genetic coding basis of psoriasis was reviewed elsewhere [4,5], the present review focused on advances in non-coding RNAs (long ncRNAs [lncRNAs] and circular RNAs [circRNAs]).

Non-coding RNAs (ncRNAs) are a class of RNAs without the capability for protein translation. However, they have diversified regulatory roles in many biological processes by affecting gene expression and cellular protein location and function. It was assumed that > 98% of the human genome is transcribed into ncRNAs, while only ~ 2% of RNAs encode proteins [6,7]. The ncRNAs provide an additional regulatory layer to balance the intracellular activity. The ncRNAs can be divided according to their length and structure: linear lncRNAs that are > 200-nucleotides long, microRNAs (miRNAs) that are ~ 22-nucleotides long, and circRNAs, a newly defined ncRNA characterized by a stable and covalently closed loop with no 5‘ and 3‘ polar ends. The ncRNAs constitute an important and complex epigenetic regulation [8], where recognizing ncRNA function gradually opens a new door to the study of complex diseases, including psoriasis, which is believed to be tightly related to epigenetic regulation [9,10]. Indeed, previous studies demonstrated that most genetic susceptibility loci identified for psoriasis fall into non-coding regions [11,12].

LncRNA expression is tissue-specific [13], which renders differentially expressed lncRNAs more likely to become disease biomarkers and potential therapeutic targets. For example, Lam et al. performed RNA sequencing (RNA-seq) analysis of biopsied skin and detected 4022 lncRNAs, among which more than one-third were novel and skin-specific. Notably, >40% of these novel lncRNAs were differentially expressed in psoriasis skin as compared with healthy skin [14].

The circRNAs are a class of evolutionarily conserved and endogenously expressed ncRNAs with tissue-specific expression patterns. Emerging research suggested that circRNA dysregulation is closely related to complex pathologies, such as cancer, neuronal disease, and autoimmune disease [15–17]. Although there are few reports on circRNAs in psoriasis research, current studies demonstrated a distinguished circRNA expression landscape of psoriasis lesions from both non-lesional and healthy skins [18,19].

Exploring lncRNA and circRNA regulation and function in psoriasis will facilitate in-depth understanding and the search for new biomarkers of this complex disease. The present review summarizes the current research progress on lncRNAs and circRNAs in psoriasis.

## LncRnas and psoriasis

LncRNAs are > 200-nucleotides long and are incapable of coding for proteins [20]. Extensive transcriptome analysis demonstrated that the majority of the mammalian transcriptome is composed of lncRNAs, which were once considered genomic transcription noise. Nevertheless, lncRNAs are associated with diverse biological functions [21]. For example, lncRNAs function as cis or trans transcription regulators [22–25], mRNA processing modulators and mRNA activity regulators by sponging miRNAs [26,27], and protein locational and functional regulators [28,29]. Despite the elucidation of potential regulatory mechanisms, the biological relevance of the vast majority of lncRNAs and their specific effects in different diseases remain uncertain.

The pivotal role of lncRNAs in skin homoeostasis and pathogenesis has attracted considerable attention. For example, the methylation modification of the lncRNA Pvt1 was critically involved in maintaining epidermal progenitor cell stemness and contributed to epidermis self‐renewal and wound healing [30]. Similarly, Li et al. identified a new lncRNA, WAKMAR1 (wound and keratinocyte migration-associated lncRNA 1), which regulated a network of protein-coding genes important for cell migration by interfering with E2F1 promoter methylation [31]. Recent studies identified numerous lncRNAs that are important in the pathology of the skin inflammatory response, for example, H19, MALAT1 (metastasis-associated lung adenocarcinoma transcript 1), NEAT1, and GAS5 [32].

### LncRNA profiles in psoriasis

Gupta et al. determined that human skin contains > 6000 lncRNAs, of which approximately 1000 are differentially expressed in psoriasis [33]. Our lab also conducted RNA-seq analysis of psoriasis, where we identified a total read of 105,136 lncRNAs, among which 1940 lncRNAs were differentially expressed between psoriasis lesions and non-psoriasis skin, and 1559 lncRNAs were differentially expressed between psoriasis lesions and healthy skin. Notably, the non-psoriasis skins also demonstrated an significant signature of 1201 differentially expressed lncRNAs when compared with healthy skin. Besides, all three comparisons shared a considerable proportion of differentially expressed lncRNAs. This proportion of lncRNAs might consistently contribute to psoriasis development both in the pre-lesion formation stage and the subsequent lesion formation stage [34]. A study using weighted gene co-expression network analysis obtained 76 lncRNA – mRNA co-expression pairs in psoriasis. Among these pairs, lncRNA AL162231.4 and *CCL27* mRNA are on the same chromosome with overlapping regions. According to the theory that a synonymous lncRNA regulates the corresponding mRNA, the author speculated that AL162231.4 might regulate CCL27 expression in psoriasis. That study proposed that identifying synonymous lncRNAs and mRNAs might be an effective approach to elucidate psoriasis pathogenesis [35].

Using an IL−22-induced HaCaT cell model, Qiao et al. determined that 1438 lncRNAs were differentially expressed, which indicated that lncRNAs are susceptible to inflammation signals in keratinocytes [36]. Furthermore, Luo et al. determined that lncRNA SPRR2C (small proline-rich protein 2C) acts as a hub gene with a critical effect on psoriasis pathogenesis by responding to IL−22 treatment. SPRR2C modulated the IL−22-stimulated HaCaT cell phenotype through the miR−330–STAT1–S100A7 axis [37]. Ahn et al. conducted WGCNA on the RNA-seq results of skin samples from psoriasis patients (pre- and post-treatment with the TNF-α inhibitor adalimumab) and healthy controls and determined that > 50% of co-expressed genes in most of the psoriasis- and treatment-associated network modules were lncRNAs; therefore, the authors speculated that lncRNAs are critical in regulation pathways involved in psoriasis pathogenesis [38]. In Table 1, we summarize several important lncRNAs that are associated with psoriasis.

**Table 1.** Summary of the currently studied lncRnas in psoriasis.

LncRNA	Expression	Tissue/cell	Function	References
PRINS	Up	Skin/keratinocyte/HaCaT	Maintenance of hyperproliferation of keratinocytes in psoriasis through control of the anti-apoptotic protein GIP3; Reduced levels of some pro-inflammatory factors in inflammatory keratinocytes by direct interaction with mRNA.	[39,40]
MIR31HG	Up	Skin/HaCaT	Promoting proliferation under NF-κB activation conditions	[41]
KLHDC7B-DT	Up	Skin/keratinocyte	Activation of JAK-STAT and MAPK signaling pathways through binding to ILF2 to promote excessive proliferation and inflammation in keratinocytes.	[42]
RP6-65G23.1	Up	Skin/HaCaT	Promotes proliferation of keratinocytes through dual activation of ERK and AKT pathways. Inhibits apoptosis by downregulating Bcl 2 and Bcl-xl.	[43]
MALAT−1	Up	DC/Macrophage/keratinocyte	Inhibits lps-induced maturation of DCs; suppresses T cell proliferation and promotes production of treg cells; and inhibits inflammatory responses by interacting with intranuclear NF-κB in lps-stimulated macrophages.	[32,44,45]
FABPSP3	Up	Skin/Keratinocyte	It maintains KMT2C overexpression through recruitment of human antigen R (HuR) thereby enhancing its downstream response to proliferation and inflammation.	[46]
AGAP2-AS1	Up	Skin/keratinocyte	Upregulation of AKT 3 by sponge miR−424- 5 p activates the AKT/mTOR pathway, thereby promoting proliferation of keratinocytes.	[47]
MSX2P1	Up	HaCaT	Binding to miR−6731-5p as a ceRNA derepresses S100A7 and elevates levels of other pro-inflammatory cytokines to promote keratinocyte proliferation and inflammation.	[36]
SPRR2C	Up	Skin/HaCaT	Enhancement of IL−22-induced proliferation and inflammatory effects on HaCaT by competitive binding with miR−330 in the miR−330/STAT1/S100A7 axis.	[37]
SLC6A14–1:1	Up	Skin	-	[48]
HSFY2–10:1	Up	Skin	Promote proliferation and inﬂammation in part by competitively binding miRNA−145 to regulate its mediated pathway.	[48]
PRANCR	Up	keratinocyte	Promote proliferation and differentiation; Absence significantly inhibits the proliferation of keratinocytes and leads to an intrinsic loss of clonogenic capacity.	[49]
LINC00941	Down	keratinocyte	Inhibits early keratinocytes differentiation	[50]
H19	Down	Skin	As a sponge for miR−766-3p, upregulates S1PR3 levels and regulates psoriatic keratinocyte proliferation and skin inflammation via the AKT/mTOR pathway.	[51]
MEG3	Down	Skin/HaCaT	Blocked regulation of inflammation and autophagy in keratinocytes by TNF-α through the PI3K/AKT/mTOR signaling pathway.	[52]
LncRNA-AL162231.4	Down	Skin	-	[35]
ST7OT	Down	Skin	-	[33]
LOC285194	Down	Skin	-	[33]
Car Intergenic 10	Down	Skin	-	[33]
NONHSAT044111	Down	Skin	-	[48]

Note: nuclear factor-κB (NF-κB); interleukin enhancer binding factor 2 (ILF2); signal transducer and activator of transcription (STAT); Janus kinase (JAK); extracellular regulated kinase (ERK); AKT serine/threonine kinase 1 (AKT); mechanistic target of rapamycin (mTOR); competitive endogenous RNA (ceRNA); Phosphoinositide 3-kinase (PI3K); Sphingosine 1-phosphate receptor 3 (S1PR3).

### Psoriasis susceptibility lncRnas

In 2005, Sonkoly et al. identified a psoriasis susceptibility-related lncRNA termed PRINS (psoriasis susceptibility-related RNA gene induced by stress), whose expression was higher in the uninvolved epidermis of psoriatic patients compared with that in both psoriatic lesions and healthy epidermis [53]. Subsequent investigations revealed that the stress state induced PRINS expression in HaCaT cells cultivated in vitro, which is essential for keratinocyte survival under stress conditions [53]. Genome-wide association studies identified TRAF3IP2 as a common susceptibility locus for psoriasis [54]. Interestingly, the antisense of TRAF3IP2, which is an lncRNA termed TRAF3IP2-AS1, is also vital in regulating psoriasis, especially in inhibiting the IL−17A-induced activation of NF-κB signalling and MAPK signalling by binding to SRSF10 and thereby blocking the subsequent recruitment of ACT1 [55]. A psoriasis-prone variant of TRAF3IP2-AS1, A4165G (rs13210247), was identified as a gain-of-function mutant with enhanced binding affinity to SRSF10. Interestingly, lncRNA E130307A14-Rik was functionally identical to TRAF3IP2-AS1. E130307A14-Rik was overexpressed by delivering E130307A14-Rik and SRSF10 lentivirus into a mouse model of psoriasis, and significantly inhibited IL−17A- and TNF-α-induced pro-inflammatory effects. Therefore, TRAF3IP2-AS1 and SRSF10 May be therapeutic targets for psoriasis [55]. Rakhshan et al. reported that a single-nucleotide polymorphism (SNP) gene, rs12826786, which is a variant of HOX transcriptional antisense RNA (HOTAIR), was associated with psoriasis risk [56]. An important lncRNA, HOTAIR is extensively involved in affecting tumour cell proliferation and apoptosis, and metastasis [57], but its involvement in psoriasis has not been well documented. Another study genotyped three HOTAIR SNPs (rs12826786, rs1899663, rs4759314) by screening the loci of key candidate SNPs in HOTAIR. Two variant loci, rs12826786 and rs4759314, were eventually demonstrated as being associated with psoriasis risk [56]. Similarly, the antisense ncRNA in the INK4 locus, termed ANRIL, which is influenced by STAT1 signalling [58], is involved in autoimmune diseases, such as multiple sclerosis and inflammatory reactions [59]. Three ANRIL variants (rs1333048, rs4977574, rs10757278) were associated with psoriasis risk. For example, the rs10757278 G allele and the rs1333048 C allele were more prevalent in psoriasis cases. However, the rs4977574 A allele was protective against psoriasis [60]. Table 2 summarizes the variant psoriasis risk-associated lncRNAs.

**Table 2.** Psoriasis susceptibility lncRnas.

lncRNA	Tissue/cell	Variants	Function	References
TRAF3IP2-AS1	Skin/keratinocyte	rs13210247	regulation of Act1 expression and IL−17A signaling by recruitment of SRSF10	[54]
ANRIL	Skin	rs1333048 rs10757278 rs4977574	activation of inflammatory factor transcripts, perhaps in response to NF-κB induction and transcription factor (YY1); rs10757278 May regulate inflammation by altering the binding site of the transcription factor STAT1 to ANRIL	[60,61]
HOTAIR	Skin	rs12826786 rs4759314	may mediate NF-κB activation and the expression of cytokines and inflammatory genes (IL−6, iNOS, TNF-α, MIP−1B)	[56,62,63]

Note: serine/arginine splicing factor 10 (SRSF10); nuclear factor-κB (NF-κB); Yin Yang 1 (YY1); signal transducer and activator of transcription (STAT); inducible nitric oxide synthase (iNOS); tumour necrosis factor-α (TNF-α); macrophage inflammatory protein−1B (MIP−1B).

### LncRnas contribute to keratinocyte dysfunction in psoriasis

**Figure 1.**
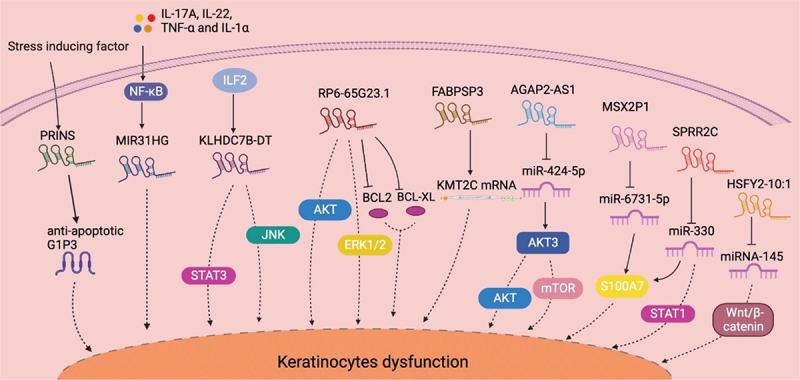
Summary of lncRNA-mediated mechanism that contributes to keratinocyte dysfunction. The stress-induced psoriasis susceptibility-associated gene PRINS promotes keratinocyte proliferation by enhancing the expression of the anti-apoptotic protein GIP3. The NF-κB signaling-dependent expression of MIR31HG directly contributes to keratinocyte proliferation. KLHDC7B-DT, which is induced by ILF2, promotes keratinocyte proliferation by activating the STAT3–JNK pathway. RP6-65G23.1 promotes keratinocyte proliferation by activating the ERK and AKT signaling pathways while inhibiting apoptosis by inactivating Bcl2 and Bcl-xl. FABPSP3 enhances keratinocyte proliferation by maintaining KMT2C mRNA stability. AGAP2-AS1 activates the AKT–Mtor pathway by acting as a ceRNA to AKT3 by sponging miR−424-5p. MSX2P1 acts as a ceRNA to S100A7 by competitively binding to miR−6731-5p, thereby accelerating keratinocyte proliferation. SPRR2C promotes keratinocyte proliferation by sponging miR−330 and activates STAT1 and S100A7 expression. HSFY2–10:1 contributes to keratinocyte proliferation by binding with miR−145, which inhibits cell proliferation and promotes apoptosis by regulating the Wnt–β-catenin signaling pathway. AKT: AKT serine/threonine kinase 1; ceRNA: competitive endogenous RNA; ERK: extracellular regulated kinase; ILF2: interleukin enhancer binding factor 2; IL−17A: interleukin 17A; JAK: Janus kinase; KMT2C: lysine methyltransferase 2C; mTOR: mechanistic target of rapamycin kinase; NF-κB: nuclear factor κB; PRINS: psoriasis susceptibility-related RNA gene induced by stress; STAT: signal transducer and activator of transcription; TNF-α: tumor necrosis factor α.

Keratinocytes are the main cell types that comprise the outmost layer of skin. As intrinsic immunity members, keratinocytes process antigens and present them to immune cells rapidly and non-specifically [64]. Keratinocyte dysregulation seriously contributes to psoriasis induction, development, and phenotype formation. Figure 1 depicts the lncRNAs that contribute to psoriatic keratinocyte dysfunction and the related mechanism.

After PRINS was associated with susceptibility to psoriasis, a subsequent study suggested a mechanism by which PRINS induces the onset of psoriatic lesions but does not maintain the disease. This led to some researchers wondering whether PRINS regulates coding proteins and thereby affects disease maintenance [53]. A subsequent study demonstrated that PRINS controls the expression of G1P3, an anti-apoptotic protein that contributes to the maintenance of keratinocyte hyperproliferation in psoriatic lesions [40].

A host gene for miR−31, lncRNA MIR31HG was first reported to promote cancer cell proliferation and invasion [65]. A study on psoriasis reported that MIR31HG was significantly upregulated in psoriasis lesions as compared with healthy skin. Knocking down MIR31HG in HaCaT cells significantly decreased KRT6 and KRT16 expression and greatly reduced the percentage of S-phase cells while increasing the percentage of G2/M-phase cells. These results suggested the role of MIR31HG in psoriasis by promoting keratinocyte proliferation. Furthermore, the authors demonstrated that MIR31HG expression appeared to be NF-κB-dependent [41].

KLHDC7B-DT was positively regulated by interleukin enhancer binding factor 2 (ILF2), and both were overexpressed in psoriatic tissues and an M5 (IL−17A, IL−22, IL−1α, oncostatin M, TNF-α) induced keratinocyte model. The investigators determined that knocking down KLHDC7B-DT in keratinocytes prohibited the M5-induced proliferation and inflammatory cytokine secretion. By contrast, overexpressing KLHDC7B-DT in HaCaT cells led to cell hyperproliferation and apoptosis prohibition. Via RNA pull-down assay and mass spectrometry detection, the authors identified a mechanism through which KLHDC7B-DT could interact with ILF2 and activate the STAT3–JNK pathway [42].

Located on chromosome 14q24.2, RP6-65G23.1 was significantly upregulated in both psoriatic lesions and an M5-induced keratinocyte model [14]. Knocking down RP6-65G23.1 in HaCaT cells notably inhibited G1/S transition and restrained cell proliferation. Further studies revealed a mechanism wherein RP6-65G23.1 dually activated the ERK and AKT signalling pathways. Moreover, RP6-65G23.1 suppressed apoptosis by downregulating BCL2 and BCL-XL [43].

LncRNA involvement in the epigenetic regulation of psoriatic keratinocytes has also received much attention. Recently, Huang et al. reported that lncRNA FABPSP3 contributed to the upregulation of lysine methyltransferase 2C (KMT2C) in psoriatic lesions by recruiting human antigen R (HuR) to maintain *KMT2C* mRNA stability. PIK3R3 overexpression promoted cell proliferation and cyclin D1 expression in psoriatic keratinocytes. The author reported that FABPSP3 promoted PIK3R3 transcription via KMT2C by regulating histone H3 lysine 4 trimethylation enrichment at the PIK3R3 promoter and histone 3 lysine 4 monomethylation at the PIK3R3\enhancer, thereby accelerating keratinocyte proliferation [46]. Furthermore, Xian et al. reported that downregulating methyltransferase-like 3 (METTL3) in psoriasis stabilized lncRNA AGAP2-AS1 by reducing its m^6^A modification; AGAP2-AS1 then functioned as a competitive endogenous RNA (ceRNA) by sponging miR−424-5p to upregulate AKT3 and activate the AKT – mTOR pathway, hence promoting keratinocyte proliferation [47].

An IL−22-induced HaCaT cell model had significantly upregulated lncRNA MSX2P1. MSX2P1 acted as a sponge of miR−6731-5p and prevented the miR−6731-5p-mediated downregulation of S100A7, thereby accelerating keratinocyte proliferation. Given that S100A7 is tightly associated with keratinocyte hyperproliferation in psoriasis, MSX2P1–miR−6731-5p biological regulation is a potential novel therapeutic target for treating psoriasis [36].

Another IL−22-responsive lncRNA, SPRR2C was one of the most highly upregulated lncRNAs in psoriasis lesions and was identified as a psoriasis hub gene. SPRR2C functioned as a ceRNA to both S100A7 and STAT1 by sponging miR−330. Notably, IL−22 induced KRT5 and KRT14 expression while inhibiting KRT1 and KRT10 expression in keratinocytes in a SPRR2C-mediated ceRNA regulation-dependent manner. Therefore, SPRR2C promotion of IL−22-induced HaCaT cell proliferation was at least mediated through the miR−330–STAT1–S100A7 axis [37].

Microarray analysis of lncRNAs in psoriatic lesions determined that 2194 lncRNAs were dysregulated, among which lnc-SLC6A14–1:1 was the most upregulated (approximately 80-fold) and NONHSAT044111 was the most downregulated (approximately 1/29-fold) [48]. One of the most significantly upregulated lncRNAs, lnc-HSFY2–10:1, was identified to competitively bind with miR−145 [48]. Importantly, miR−145 inhibits keratinocyte proliferation and promotes apoptosis by regulating the Wnt–β-catenin signalling pathway [66]. This information suggested a potential role of lnc-HSFY2–10:1 in psoriasis development, but its effect on keratinocytes remains unknown.

A CRISPR interference (CRISPRi) screen of lncRNAs in human keratinocytes identified progenitor renewal-associated ncRNA (PRANCR) as an important regulator of epidermal homoeostasis. Depleting PRANCR significantly inhibited keratinocyte proliferation and resulted in the intrinsic loss of clone formation capability. In contrast, there was no difference in apoptosis induction after PRANCR had been depleted [49].

According to the rules of ceRNA regulation, the downregulated lncRNAs in psoriasis could not protect the target mRNAs from miRNA-mediated degradation. Therefore, most of them were related to the coding genes that were also downregulated in psoriasis. For example, lncRNA H19 protected S1PR3 from miR−766-3p-mediated degradation. In psoriatic skins, miR−766-3p overexpression was correlated with the downregulation of both lncRNA H19 and S1PR3 [63]. Using a keratinocyte model, He et al. reported that miR−766-3p activated the cell survival-related AKT – mTOR signalling; however, the signalling was inactivated by H19. The import of exogenous miR−766-3p counteracted the inhibitory effect of H19 on keratinocyte proliferation in a S1PR3-dependent manner [51].

Encoded on chromosome 12, LINC00941 is a key regulator of human epidermal homoeostasis. LINC00941 is most highly expressed in undifferentiated progenitor keratinocytes and its abundance decreases significantly during cell terminal differentiation. The finding suggested an important role for LINC00941 in suppressing premature keratinocyte differentiation. Interestingly, LINC00941 and SPRR5 are inversely regulated in epidermal differentiation. Moreover, more than half of the LINC00941-induced genes were repressed by SPRR5. That study reported an interesting pair of lncRNAs with opposite roles in keratinocyte differentiation [50].

### LncRnas regulate keratinocyte inflammation in psoriasis

**Figure 2.**
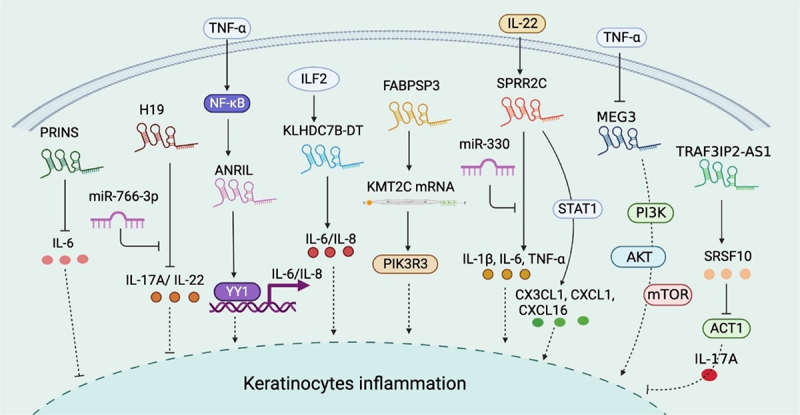
Summary of lncRNA-mediated mechanism that contributes to keratinocyte inflammation. PRINS targets IL6 mRNA to suppress inflammation. H19 reduces the expression of inflammatory cytokines such as IL−17A and IL−22, while miR−766-3p impairs the H19-mediated regulation. The NF-κB-dependent expression of ANRIL forms a functional complex with transcription factor YY1 and binds to the IL−6 and IL−8 promoters to activate their expression. ILF2 enhancement of IL−6 and IL−8 expression in psoriasis is dependent on KLHDC7B-DT. FABPSP3 enhances KMT2C mRNA stability, which activates inflammatory signaling pathways and promotes the secretion of inflammatory mediators by enhancing PIK3R3 transcription. SPRR2C promoted the expression of inflammatory cytokines, such as IL−1β, IL−6, and TNF-α, in an IL−22-induced cell model. SPRR2C also enhanced CX3CL1, CXCL1, and CXCL16 expression by activating the STAT1 signaling pathway. However, miR−330 negatively affected SPRR2C-mediated regulation. The TNF-α-dependent expression of MEG3 aggregated keratinocyte inflammation by activating PI3K–AKT–Mtor signaling. TRAF3IP2-AS1 binds to SRSF10 to block the recruitment of ACT1 and thereby inhibit the IL−17A-induced pro-inflammatory effects. YY1: Yin Yang 1; PIK3R3: phosphoinositide 3-kinase regulatory subunit 3; CX3CL: the C-X3-C motif chemokine ligand; CXCL: chemokine (C-X-C motif) ligand; PI3K: phosphoinositide 3-kinase; MAPK: mitogen-activated protein kinases.

Excessive keratinocyte proliferation is closely related to inflammation conditions, where a substantial proportion of lncRNAs that have been identified to regulate keratinocyte proliferation is also involved in regulating inflammation. Figure 2 summarizes their regulation mechanisms. Apart from affecting keratinocyte proliferation and differentiation, PRINS reduced IL−6, IL−8, and CCL−5 secretion from keratinocytes, but did not affect the expression of other inflammatory factors, such as IL−1α, IL−1β, and TNF-α [39]. Furthermore, PRINS demonstrated very high binding affinity to *IL6* mRNA, and the specific interaction disrupted IL−6 translation and secretion in normal human epidermal keratinocytes (NHEKs) [39]. As IL−6 and IL−8 are actively involved in the inflammatory response in psoriasis [67], PRINS has a potential inhibitory effect on psoriasis inflammation.

Introduced in the previous section, ANRIL is induced by inflammatory stimuli such as TNF-α in an NF-κB-dependent manner. In contrast to PRINS, ANRIL activates IL−6 and IL−8 transcription through interaction with the transcription factor Yin Yang 1 (YY1), which functionally binds the IL−6 and IL−8 promoters upon activation [61].

As previously described, KLHDC7B-DT was significantly upregulated in psoriatic tissues, where it acts as an inflammation promoter. For example, knocking down KLHDC7B-DT greatly weakened the M5-induced inflammatory response in keratinocytes. Moreover, ablating KLHDC7B-DT significantly reversed the ILF2-mediated induction of IL−6 and IL−8 in keratinocytes [42].

The lncRNA FABPSP3 is an underlying cause of KMT2C overexpression in psoriasis by maintaining KMT2C stability. In addition to promoting keratinocyte proliferation, KMT2C is a well-documented inflammatory responder via the induction of PIK3R3, which subsequently mediates Akt – NF-κB pathway activation and results in the production of inflammatory mediators, such as IL−6, IL−8, CCL20, and S100A9 [46].

In an IL−22-induced keratinocyte model, SPRR2C contributed to the generation of inflammatory cytokines, including IL−1β, IL−6, and TNF-α, and this function is tightly related to its upregulation on STAT1 and S100A7. However, miR−330 largely blocked the IL−22–SPRR2C axis-induced inflammatory effects. Additionally, knocking down SPRR2C in human primary keratinocytes suppressed IL−22-induced elevated levels of CX3CL1, CXCL1, and CXCL16, probably through STAT1 signalling [37].

Conversely, H19, which is significantly downregulated in psoriasis epidermis, reduced the expression of inflammatory cytokines in IL−17A-, IL−22- and M5-induced keratinocyte models, while the addition of miR−766-3p to these cell models attenuated H19 regulation of inflammation [51].

Tang et al. reported that lncRNA MEG3 was downregulated in psoriatic skin and acted as a target molecule of TNF-α. Similar downregulation of MEG3 was detected in both a TNF-α induced keratinocyte model and TNF-α induced mouse model. The author demonstrated that recovering MEG3 expression blocked TNF-α regulation on keratinocytes, which included enhancing inflammation and suppressing autophagy, likely via PI3K – AKT–mTOR signalling [68].

### LncRNA regulates immune cell functions in psoriasis

Psoriasis was considered an immune-mediated inflammatory skin disease, where diverse immune cell types that infiltrate the skin tissue contribute to the inflammation cascade and subsequent phenotype formation. Currently, there are relatively few reports on the regulating roles of lncRNA in immune cell functions in psoriasis. However, when referring to another disease such as cancer, numerous studies indicated that lncRNAs were associated with the development and functions of various immune cell types. We believe that more efforts should be made to explore lncRNA regulation of psoriasis-associated immune cells.

A recent report using integrated analysis identified several immune-related lncRNAs as diagnostic biomarkers of psoriasis. For example, LINC01137, CCDC18-AS1, and CARMN were positively correlated with activated memory CD4^+^ T cells, myeloid dendritic cells (DC), neutrophils, M1 macrophages, and T follicular helper (Tfh) cells and were negatively correlated with T regulatory cells (Treg). LINC01215, MAPKAPK5-AS1, TPT1-AS1, EPB41L4A-AS, and LINC01214 were negatively correlated with activated memory CD4^+^ T cells, activated myeloid DC, neutrophils, M1 macrophages, and Tfh cells and were positively correlated with Treg [69]. In this section, we summarize several important immune-related lncRNAs, although some of them were not reported in relation to psoriasis, which might suggest implications for future psoriasis studies.

HOTAIR is an extensively explored lncRNA with a notable role in regulating immune cell functions. In lipopolysaccharide (LPS)-induced macrophages, HOTAIR critically mediated NF-κB signalling activation and the subsequent secretion of inflammatory cytokines such as TNF-α, IL−6, macrophage inflammatory protein−1B (MIP−1B), and inducible nitric oxide synthase (iNOS) [70]. Crucially, SNPs in the HOTAIR locus are associated with psoriasis risk [56]. These SNPs may influence susceptibility to psoriasis by regulating inflammatory factor levels in immune cells. However, there remains a lack of sufficient evidence for the association between HOTAIR and psoriasis.

On the contrary, MALAT1 was negatively associated with LPS-induced innate immune responses in human macrophages. Cells with small interfering RNA (siRNA)-mediated knockdown of MALAT1 had significantly increased TNF-α and IL−6 production in conjunction with LPS stimulation. Mechanistically, MALAT1 exerted an inhibitory effect on inflammation by interacting with NF-κB in the nucleus, thereby inhibiting its DNA binding activity [44]. Notably, MALAT1 mediated the inflammation progress in a high-glucose environment by upregulating IL−6 and TNF-α expression in endothelial cells [71]. This implied that the anti- or pro-inflammatory effects of MALAT1 differ between cell lines and stimulations. Wu et al. used an LPS-stimulated DC model and reported that MALAT1 overexpression prevented IL−6, IL−12, and interferon (IFN)-γ induction, whereas it promoted the secretion of the anti-inflammatory cytokine IL−10. Furthermore, MALAT1-suppressed DC had markedly decreased expression of co-stimulatory molecules CD80, CD86, and MHCII, indicating that MALAT1 inhibited the maturation of LPS-induced DC. Moreover, T cells co-cultured with LPS-treated MALAT1-overexpressing DC had significantly reduced proliferation capacity and increased percentages of Treg. Therefore, MALAT1 promoted the transition of LPS-stimulated DC to a tolerant phenotype, which led to effector T cell inactivation [45]. The DC-specific intercellular adhesion molecule 3 grasping non-integrin (DC-SIGN) is an intrinsic immune receptor expressed mainly by DC and macrophages and is involved in immunosuppressive maintenance and immune tolerance. MALAT1 overexpression in DC promoted DC-SIGN expression by functioning as a miR−155 sponge [72]. That study highlighted the role of MALAT1 in mediating DC immune suppression. Psoriasis tissues had increased MALAT1 levels [32]; nevertheless, the exact functions require clarification.

Analysis of data from the Immunological Genome Project (ImmGen) compendium of immunocyte gene expression determined that lncRNA FLICR (Foxp3 long intergenic ncRNA) was specifically expressed in Treg and co-occurred with Foxp3 during cell differentiation [73]. FLICR negatively regulated Foxp3, and this effect was particularly marked in IL−2 deficiency conditions. Analysis of transposase accessible chromatin (ATAC) high-throughput sequencing inferred that FLICR affected chromatin accessibility in the Foxp3 conserved non-coding sequence 3 (CNS3, which is important for Treg differentiation) accessible region 5 (AR5) through a local cis-inhibitory manner. Therefore, FLICR in cis targets the Foxp3 CNS3/AR5 region, thereby inhibiting Treg activity, and this effect is more pronounced in the absence of IL−2 [73]. Considering that Foxp3 stability is essential for maintaining Treg dynamic homoeostasis to prevent autoimmunity, the inhibitory effect of FLICR on Foxp3 May exacerbate psoriasis, which requires further elucidation.

The IL−17-derived T helper cells (Th17 cell) are important in the pathogenesis of autoimmune diseases, including psoriasis. The peripheral Th cell-expressed lncRNA NONHSAT079547.2 was positively correlated with IL−17 expression in Hashimoto’s thyroiditis. Further investigation suggested a mechanism by which NONHSAT079547.2 acts as a ceRNA to *IL17* mRNA, where it competitively binds with miR−4716-5p, which could directly target the *IL17* mRNA sequence [74].

### LncRnas link psoriasis with cardiometabolic diseases

Psoriasis is considered a systemic disorder rather than a skin-exclusive disease, where a particularly convincing amount of evidence indicated that psoriasis is associated with cardiovascular diseases [75,76]. Psoriasis-related systemic inflammation has been identified as an independent risk factor for cardiometabolic disease [77]. The genetic and molecular pathways shared by psoriasis and cardiometabolic diseases were well reviewed elsewhere [78,79]. However, existing reports on the shared lncRNAs between the two disease types remain highly limited. Herein, we summarize the shared lncRNAs studied in both psoriasis and cardiometabolic diseases.

The NF-κB-dependent ANRIL, which may serve as a psoriasis biomarker, is located in the INK4 locus, a well-defined genetic risk locus associated with coronary artery disease. Furthermore, it was suggested that the NF-κB-dependent ANRIL regulates inflammatory responses in coronary artery disease by activating IL−6/8 transcription. H19, which regulates IL−17A expression in psoriasis, is also dysregulated in cardiometabolic diseases. Specifically, H19 was enriched in cardiac and vascular tissue, and a multifunctional role of H19 in cardiometabolic diseases was suggested. H19 polymorphisms were associated with ischaemic stroke, and ischaemic stroke patients have higher levels of circulating H19 [80,81]. Furthermore, H19 regulated cardiomyocyte function and was affected in the progression of cardiac remodelling [82,83]. Moreover, H19 is widely involved in other cardiometabolic diseases, such as pulmonary hypertension, aneurysmal disease, haemorrhagic stroke, hypoxic-ischaemic encephalopathy, myocardial infarction, and coronary artery disease, which was well-reviewed by Busscher [84]. In addition to mediating pro-inflammation reactions of macrophages in psoriasis [70], HOTAIR also affects adipocyte differentiation and function, where it is specifically expressed in gluteal but not abdominal subcutaneous adipose tissue, and may be involved in determining the metabolic properties of gluteal compared with abdominal adipocytes. Therefore, HOTAIR might be associated with cardiometabolic risk by regulating peripheral body fat development [85]. In psoriasis, MEG3 might act as an antagonist to inflammatory signals [68]. However, MEG3 methylation modification might induce disorders, for example, the higher blood levels of MEG3 methylation in children due to prenatal lead exposure are more likely to generate rapid adiposity gain, which is a risk factor for childhood obesity and cardiometabolic diseases in adulthood [86]. Furthermore, MALAT1 negatively regulates the innate immune responses in psoriasis [44,45,72]. However, MALAT1 aggravated cardiomyocyte scorch death in diabetic cardiomyopathy by targeting NLRP3, a well-characterized inflammasome [87].

We believe that more efforts should be made to explore the dysregulated lncRNAs common to both psoriasis and cardiometabolic diseases, which may aid the clarification of the mechanistic association between the two disease types.

## CircRnas and psoriasis

Unlike most linear mRNAs and lncRNAs with 5‘ N7-methylguanosine (m^7^G) caps and 3‘ polyadenylated tails, circRNAs are covalently closed single-stranded RNAs (ssRNAs) also present in eukaryotes [88,89]. Although circRNAs were known of for many years, their roles were largely underestimated until the development of RNA-seq technologies and the further study of their molecular aspects [90]. In skin diseases, circRNAs were associated with melanoma occurrence, progression, and tumour malignant behaviours, which included metastasis and invasion [91,92]. RNA-seq identified many differentially expressed circRNAs in psoriasis, and some circRNAs may be candidate biomarkers or involved in psoriasis pathogenesis [19,93]; several circRNAs may contribute to the development and pathophysiology of hypertrophic scars [94,95]. In this section, we review the current understanding of circRNA biology and circRNA functions in relation to psoriasis.

### Characterization of circRnas

CircRNAs are generated by the back-splicing of precursor mRNA (pre-mRNA), which competes with canonical mRNA splicing to produce a linear RNA. CircRNAs can be divided into three main types based on biogenesis from different genomic regions: exonic circRNAs that consist of a single exon or multiple exons and account for most of the known circRNAs [96], intronic circRNAs that only contain introns and may rely on a consensus motif containing a specific base distribution [97], and exon-intron circRNAs, which contain sequences derived from both exons and introns (Figure 1) [98].

**Figure 3.**
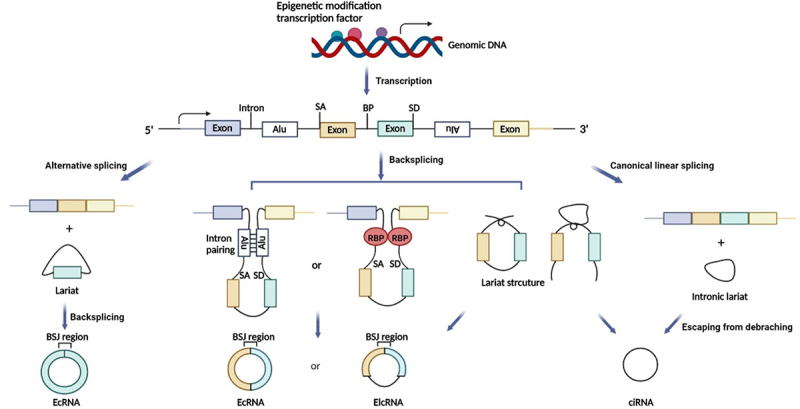
The biogenesis of circRnas. SA, Splice acceptor site; BP, branch point; SD, splice donor site; BSJ, back-splicing junction; RBP, RNA-binding protein; EcRNA, exonic circRNA; EIcRNA, exon-intron circRNA; ciRNA, intronic circRNA.

CircRNAs are primarily derived from canonical splice sites, and their expression is determined by pre-mRNA transcription levels [99,100]. CircRNA biogenesis can be modulated by transcription factor activity and epigenetic modifications, such as methylation [101–103]. Additionally, circRNA processing events are determined by the regulation of circularization, which involves three main hypotheses: lariat-driven circularization, in which the looping structure of the intron sequences flanking the downstream splice donor site and upstream splice acceptor site brings these sites into close proximity; intron pairing-driven circularization, in which looping can be mediated by base pairing between inverted complementary repeat sequences (e.g. Alu elements); and RNA-binding protein (RBP)-mediated circularization, in which RBPs are dimerized and bind to specific motifs in the flanking introns to facilitate back-splicing (Figure 3) [104,105].

**Figure 4.**
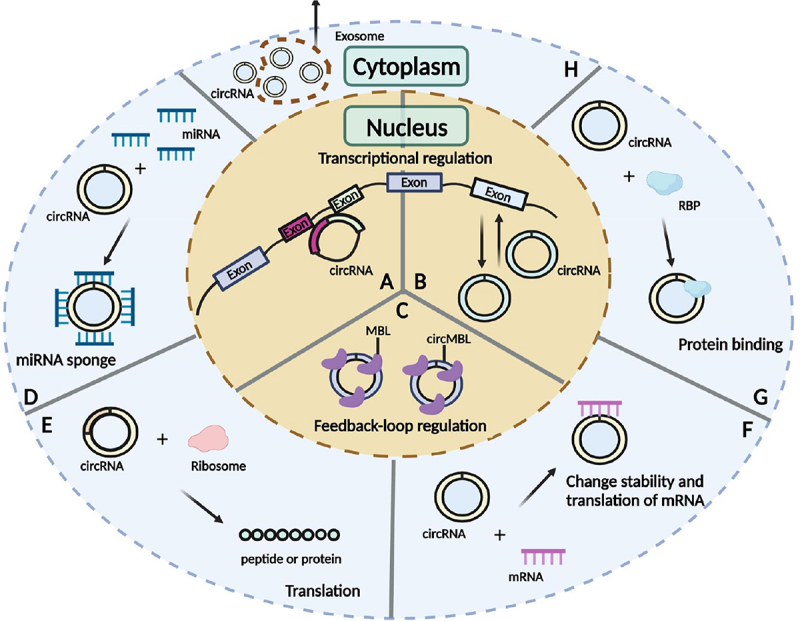
The functions of circRnas. (A) Modulate transcription. (B) Regulate splicing. (C) Sponge proteins. (D) Sponge miRnas. (E) Code peptide or protein. (F) Interact with mRnas. (G) Bind to proteins. (H) Packaged into exosomes. MBL, Multifunctional protein muscleblind; RBP, RNA-binding protein.

Increasing evidence suggested that circRNAs modulate gene expression at the transcriptional level and/or post-transcriptional processing, regulate splicing, sponge miRNAs or proteins, and code peptides or proteins [88,89,106] (Figure 4). CircRNAs binding to miRNAs can act as ceRNAs, thereby modulating the ability of miRNAs to target mRNAs [107]. Additionally, circRNAs can also bind to other RNA molecules, such as mRNAs, to regulate the stability and functions of these RNAs [108]. CircRNAs can bind to specific RBPs and thereby influence the functions of these RBPs [109]. Although most circRNAs are present in the cytoplasm [89,90,105], some can be found in the nucleus of human cells via generation from processed intron lariats or back-splicing with retained introns [89,90,104,105]. CircRNAs abundantly expressed in the nucleus are more likely to modulate transcription and splicing [110]. Many circRNAs have been validated to carry open reading frames (ORFs) and may encode peptides or proteins in an internal ribosome entry site (IRES) or N6-methyladenosine (m^6^A)-dependent manner [110–113]. In particular, circRNAs are abundant in exosomes, which means they are signal transmitters [114].

### CircRNA roles in psoriasis

CircRNAs are key in regulating cell proliferation, the immune system, and the inflammatory response [89,115,116]. An increasing number of studies have proven that many circRNAs are abnormally expressed between psoriatic lesions and normal healthy skin tissues [18,19,117–119], and might be involved in psoriasis occurrence and development through a variety of molecular mechanisms, which provide more evidence that circRNAs are potential novel therapeutic targets and biomarkers for psoriasis diagnosis, prognosis, and therapy.

Many attempts have been made to discover psoriasis biomarkers; however, no specific marker can accurately screen disease severity and treatment efficacy. CircRNAs can exist stably in cells and body fluids, such as serum and plasma, which indicates their potential as diagnostic biomarkers [120]. Several studies profiled circRNA expression in serum, peripheral blood mononuclear cells (PBMC), Treg, or lesional skin from psoriatic patients (Table 3).

**Table 3.** Expression and function of circRnas in psoriasis.

CircRNA	Research model	Expression change	Targeting miRNAs or genes	Mechanisms	Ref
hsa_circ_0056856	serum and PBMC	up	miR−1301-3p, miR−140-3p, miR−93-3p, miR−19b−3p, and miR−187-5p	Participate in epigenetic regulation in psoriasis through a ceRNA mechanism; Acting as a biomarker for psoriasis diagnosis, severity, and therapeutic response evaluation	[121]
hsa_circ_0003689, chr4:121675708|121732604, and hsa_circ_0003718	plasma and S-MSC of psoriatic lesions	up	-	Associated with the pathogenesis of psoriasis	[93]
hsa_circ_0003738	Treg	up	miR−562/IL17RA, miR−490-5p/IFNGR2	Regulate the impaired function of Tregs in the pathogenesis of dysfunctional Tregs	[122]
hsa_circ_0061012	psoriatic lesions	up	miR−194-5p/GAB1	Accelerate IL−22-induced proliferation and metastasis in keratinocytes through a miRNA sponge mechanism in psoriasis	[19,117]
Has_circ_ZRANB1	lesional skin	up	-	Show as a promising biomarker for discriminating AD from psoriasis	[18]
Has_circ_0001946	lesional skin	down	-	Show as a promising biomarker for discriminating AD from psoriasis	[18,118,119]
chr2:206992521|206994966	S-MSC of psoriatic lesions	down	-	Affect the activity of T lymphocytes in local lesions by influencing their cytokine secretion; mediate the roles of S-MSCs in the pathogenesis of psoriasis	[123]

Note: peripheral blood mononuclear cell (PBMC); skin mesenchymal stem cells (S-MSCs); regulatory T cells (Tregs); atopic dermatitis (AD).

These studies suggested that some circRNAs might be promising psoriasis screening biomarkers, while others suggested circRNAs could be used for psoriasis diagnosis. In the future, combining circRNA expression detection with clinical psoriasis diagnosis processing might improve psoriasis diagnostic accuracy as compared with traditional diagnosis approaches.

Some researchers focused on the roles of circRNAs in psoriasis. hsa_circ_0061012, hsa_circ_RAB3B, hsa_circ_IGF1R, hsa_circ_0060531, and hsa_circ_0003738 acted as miRNA sponges in psoriasis pathogenesis [117,122,124–126].

In psoriasis, circRNAs are crucial in keratinocyte motilities, including proliferation, invasion, and migration [117,122,124–126]. For example, hsa_circ_0061012 was involved in IL−22-induced HaCaT cell proliferation, migration, and invasion by sponging miR−194-5p [117]. IL−22 treatment in HaCaT cells upregulated hsa_circ_IGF1R expression, and similar to hsa_circ_0061012, circ-IGF1R promoted HaCaT cell proliferation, migration, and invasion and suppressed HaCaT cell apoptosis by targeting the miR−194-5p – CDK1 axis [125]. Furthermore, hsa_circ_0060531 was also significantly increased in IL−22-stimulated HaCaT cells, and knocking down hsa_circ_0060531 suppressed IL−22-induced HaCaT cell proliferation, migration, and inflammation by modulating the miR−330-5p – GAB1 pathway [126]. On the contrary, some circRNAs exerted opposite effects. For example, hsa_circ_RAB3B was decreased in IL−22-treated HaCaT cells, which inhibited their proliferation, migration, invasion, and cell cycle progression and induced apoptosis partly by modulating the miR−1228-3p – PTEN axis [124].

Treg are a subgroup of immunosuppressive CD4^+^ T cells that dominantly mediate immune tolerance and maintain immune homoeostasis [127]. In psoriasis, impaired and dysfunctional Treg result in the abnormal proliferation of effector T cells, which disturbs immune homoeostasis and was associated with psoriasis development. hsa_circ_0003738 was obviously stimulated in psoriatic Treg [122]. Importantly, knocking down hsa_circ_0003738 in psoriatic Treg restored their suppressive functions by inhibiting the secretion of the pro-inflammatory cytokines IL−17A and IFN-γ [122]. Moreover, hsa_circ_0003738 can harbour miR−562 to relieve the suppression of the miR−562 target gene IL−17RA, thereby promoting IL−17A signalling activation in psoriatic Treg. Moreover, hsa_circ_0003738 sponged miR−490-5p and released the inhibition of the target gene *IFNGR2*, and promoted IFN-γ signalling pathway activation in psoriatic Treg.

Yang et al. verified that hsa_circ_0004287 was enriched in PBMCs from psoriasis patients [128] and protected against psoriasis inflammation. In macrophages, hsa_circ_0004287 alleviated the stability of its host gene *MALAT1* by competitively binding to growth factor 2 mRNA-binding protein 2 (IGF2BP2) in a m^6^A-mediated RNA modification-dependent manner [128]. IGF2BP3 regulated mRNA stability by serving as a m^6^A reader [129], and m^6^A sites were found within full-length MALAT1 [130]. This finding suggested that circRNAs might modulate their host gene expression via RNA modification, which warrants further studies.

Finally, we hypothesize that certain circRNAs in various psoriasis-related functional cells may participate in psoriasis occurrence and progression by sponging numerous miRNAs and/or modulating host gene expression, which may present a new theoretical framework for additional research on circRNA functions in psoriasis. Furthermore, it was suggested that circRNAs might become the clinical biomarkers of psoriasis. However, this hypothesis is far from convincing. Therefore, more extensive and intensive research is needed to reveal the association between circRNAs and psoriasis, and the underlying molecular mechanism.

## Conclusion

As a chronic recurrent skin disease, the complex mechanism of psoriasis is unclear, where it is a serious skin disease with multiple participating and influencing cells and factors. Genetic alteration may be a key factor among the initiating, maintaining, and relapsing elements of psoriasis [4,12]. Research on the non-coding genes, which comprise the majority of the human genome, will aid in-depth understanding of the background mechanism of various complex and incurable diseases, including psoriasis.

According to the lncRNA profiles of psoriasis reported by different laboratories, approximately one-fifth of lncRNAs are differentially expressed [14,34], which indicates that lncRNAs are profoundly involved in psoriasis pathogenesis. Current research of lncRNAs in psoriasis mainly focuses on their regulation of keratinocyte proliferation, differentiation, and inflammation, and the functions of immune cells, including DC, macrophages, Treg, and Th17 cells. Several lncRNAs might act as psoriasis biomarkers and/or therapy targets, such as the IL−22-responsive SPRR2C [37], psoriasis susceptibility gene PRINS [39,40], NF-κB-dependent ANRIL [61], IL−17A-promoting H19 [51], and the innate immune response-related HOTAIR [70] and MALAT1 [44,45,71]. However, considering that the functions of most differentially expressed lncRNAs in psoriasis remain unknown, substantial efforts should be made to systematically screen the role of these differential lncRNAs in psoriasis in conjunction with the diversity of lncRNA regulation modes.

In this review, we also introduced the characterization of circRNAs, a novel ncRNA, and summarized the currently researched circRNAs in psoriasis. These reports demonstrated that the differential circRNAs in psoriasis affected keratinocyte motilities [117,124–126], Treg [131], macrophage functions, and RNA modifications of host genes [128]. Notably, hsa_circ_0004287 reduced the stability of *MALAT1* [128], an important immune-suppressive gene in psoriasis [44,45,72]. Further studies are needed to elucidate the role of the hsa_circ_0004287–MALAT1 axis in psoriasis.

Recent studies also indicated a considerable number of reports on the existence of stable, functional small peptides (micropeptides) translated from ncRNAs [132,133]. These findings added another layer of lncRNA and circRNA regulating functions. We look forward to more psoriasis-related research reports that can reveal the innovative functions and mechanisms of lncRNAs and circRNAs, which may enable a more comprehensive and in-depth understanding of this complex skin disease.
